# Supplementary material for: MetaRibo-Seq measures translation in microbiomes
Source: Nat Commun. 2020 Jun 29;11:3268. doi: 10.1038/s41467-020-17081-z (PMC7324362; doi:10.1038/s41467-020-17081-z)
Supplement: Supplementary file 10 — Supplementary Data 7 [file 41467_2020_17081_MOESM10_ESM.zip › File2/Confidence_High_Taxonomy/222274_out.krona.html]

Javascript must be enabled to view this page.

members
magnitude
magnitudeUnassigned
count
unassigned
taxon
rank

222274\_out

2

2
superkingdom
2

phylum
1239
2

2
186801
class

2
186802
order


SRS013521\_contig\_number\_contig-100\_15144.15144
species
1897045
1

216572
family
1

1
459786
genus

SRS014235\_contig\_number\_contig-100\_41094.83096
